# Diagnostic and prognostic signatures of glomerular membrane dysregulation in immune nephropathies

**DOI:** 10.3389/fimmu.2026.1800258

**Published:** 2026-03-23

**Authors:** Chengkun Wu, Keng Ye, Zigui Zheng, Yanfang Xu, Zhimin Chen

**Affiliations:** 1 Department of Nephrology, Blood Purification Research Center, The First Affiliated Hospital, Fujian Medical University, Fuzhou, China; 2 Research Center for Metabolic Chronic Kidney Disease, The First Affiliated Hospital, Fujian Medical University, Fuzhou, China; 3 Department of Nephrology, National Regional Medical Center, Binhai Campus of the First Affiliated Hospital, Fujian Medical University, Fuzhou, China; 4 School of Medicine, Nankai University, Tianjin, China; 5 Central Laboratory, The First Affiliated Hospital, Fujian Medical University, Fuzhou, China

**Keywords:** biomarkers, glomerular membrane homeostasis, immune infiltration, immunonephropathy, regulated cell death

## Abstract

**Background:**

Immunonephropathy, encompassing disorders such as anti-neutrophil cytoplasmic antibody (ANCA)-associated vasculitis (AAV), focal segmental glomerulosclerosis (FSGS), minimal change disease (MCD), and membranous nephropathy (MN), is characterized by immune-mediated glomerular injury leading to progressive renal dysfunction. Despite advances in clinical characterization, the precise molecular mechanisms underlying glomerular damage remain poorly understood.

**Methods:**

Gene expression profiles from the Gene Expression Omnibus (GEO) database were analyzed to identify plasma membrane homeostasis-related genes differentially expressed between immunonephropathy and healthy controls. Functional enrichment analyses were performed to investigate the biological pathways involved in disease progression. Least absolute shrinkage and selection operator (LASSO) regression and support vector machine (SVM) algorithms were used to identify diagnostic signature genes. Immune infiltration analysis and correlation analyses were further conducted to evaluate the associations between characteristic genes and clinical parameters, including estimated glomerular filtration rate (eGFR), proteinuria, and serum creatinine.

**Results:**

Differentially expressed plasma membrane homeostasis-related genes were identified in immunonephropathy. Functional enrichment analyses revealed significant enrichment of immune- and metabolism-related pathways. An eight-gene diagnostic signature consisting of IPMK, TP53, SLC40A1, NCOA4, SLC39A7, KEAP1, TNIP1, and SAT1 demonstrated high diagnostic accuracy. Immune infiltration analysis further revealed disease-specific immune profiles. Correlation analyses showed that KEAP1 and SLC40A1 were positively associated with proteinuria, whereas TNIP1 and TP53 were significantly associated with impaired renal function.

**Conclusions:**

Necroptosis, pyroptosis, and ferroptosis may be involved in glomerular injury in immunonephropathy. The identified characteristic genes provide insight into the molecular landscape of immunonephropathy and may serve as potential biomarkers for disease characterization.

## 1 Introduction

Immunonephropathy, encompassing disorders such as anti-neutrophil cytoplasmic antibody (ANCA) - associated vasculitis (AAV), focal segmental glomerulosclerosis (FSGS), minimal change disease (MCD), and membranous nephropathy (MN), is characterized by immune-mediated glomerular injury that leads to progressive renal dysfunction and, without effective intervention, end-stage renal disease (ESRD) (1–4). The glomerular filtration barrier, a complex structure composed of endothelial cells, podocytes, and the glomerular basement membrane, is critical for maintaining selective permeability and proper filtration. Disruption of this barrier—often involving the dysregulation of glomerular homeostasis—leads to proteinuria, a hallmark of kidney injury in these immune-mediated diseases (5). While the clinical manifestations of these subtypes are well-documented, the precise molecular drivers of glomerular damage across the disease continuum remain incompletely understood.

Emerging evidence highlights regulated cell death (RCD) as a central pathogenic factor in the loss of glomerular cell integrity and mesangial stability. Recent mechanistic studies have demonstrated that necroptosis, driven by the activation of receptor-interacting protein kinases (RIPK1/RIPK3) and mixed lineage kinase domain-like protein (MLKL), triggers membrane rupture and the release of damage-associated molecular patterns (DAMPs), thereby amplifying inflammation in glomerular diseases (6, 7). Similarly, pyroptosis involves inflammasome-mediated activation of gasdermin proteins, which form membrane pores in podocytes and endothelial cells, leading to pro-inflammatory cytokine release (8). Furthermore, ferroptosis—an iron-dependent process characterized by lipid peroxidation and reactive oxygen species (ROS) accumulation—has been shown to destabilize plasma membranes and cause oxidative injury to the filtration barrier (9, 10).

Despite these advances, studies in immune-mediated kidney diseases have largely interrogated RCD programs in isolation, often emphasizing a single pathway (e.g., pyroptosis, necroptosis, or ferroptosis) within a specific model or disease subtype. Increasing evidence indicates that these lytic RCD modalities converge on plasma membrane injury and rupture, and that membrane-repair mechanisms (e.g., ESCRT-III) can transiently preserve membrane integrity and shape inflammatory outputs (11, 12). Together, this suggests that dysregulated plasma membrane homeostasis may represent an integrated axis driven by overlapping RCD signaling. However, the extent to which necroptosis, pyroptosis, and ferroptosis jointly contribute to membrane instability and glomerular injury across immunonephropathy subtypes remains poorly defined, warranting systematic cross-pathway molecular characterization to support robust biomarker discovery (8, 13).

To address these gaps, this study utilizes an integrative approach, combining multi-center transcriptomic data with advanced machine learning algorithms to systematically characterize the molecular features of glomerular membrane dysregulation. By employing least absolute shrinkage and selection operator (LASSO) and support vector machine-recursive feature elimination (SVM-RFE) models, we sought to identify a robust diagnostic gene signature across various immunonephropathy subtypes. Furthermore, we aimed to evaluate the associations between these molecular signatures and key clinical parameters—such as estimated glomerular filtration rate (eGFR) and proteinuria—while exploring their potential impact on the glomerular immune microenvironment. Ultimately, this work seeks to elucidate the molecular associations between glomerular membrane instability and regulated cell death, providing a foundation for future functional studies on precision diagnostics.

## 2 Materials and methods

### 2.1 Data source

**Figure 1:**
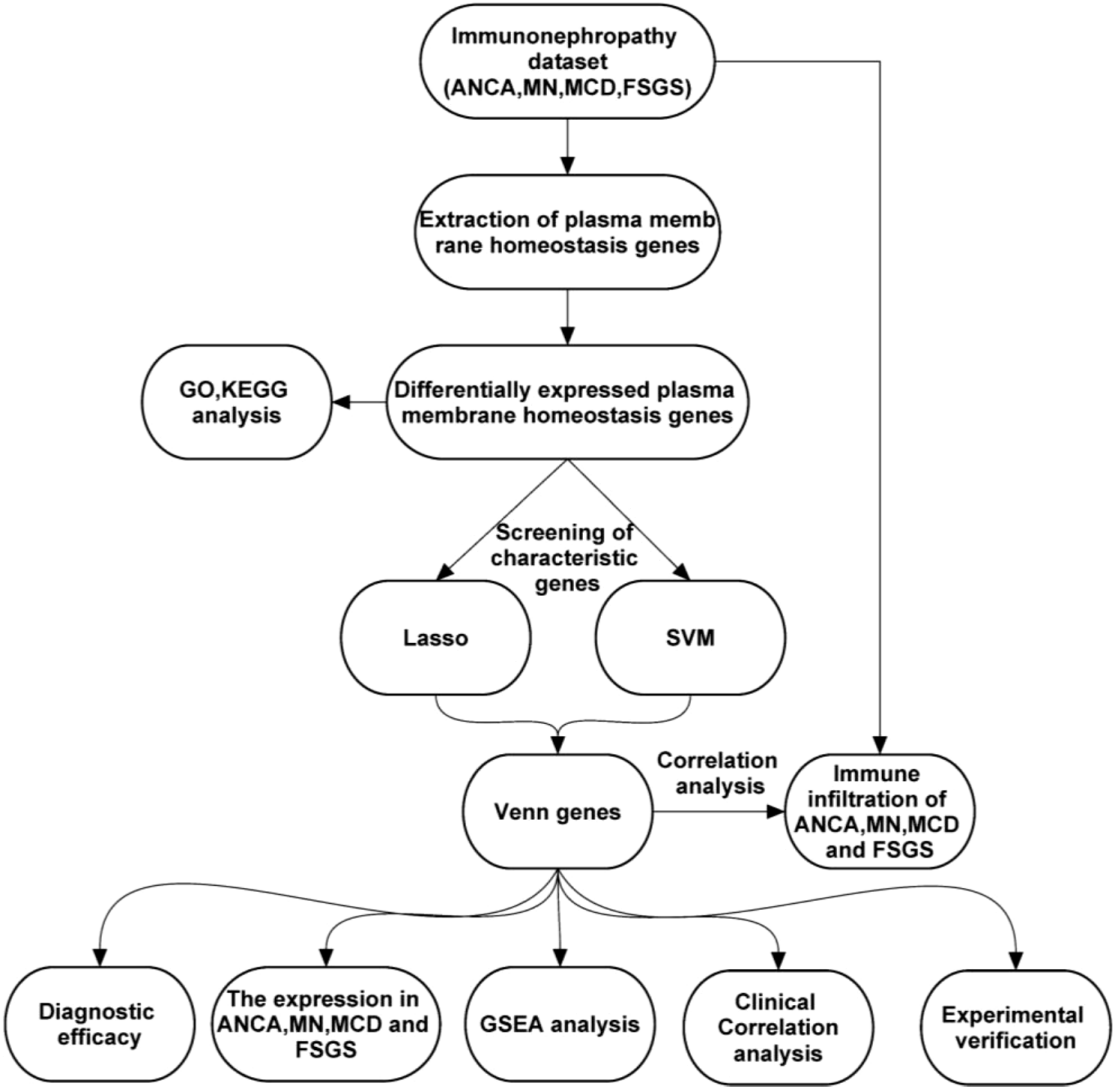
Workflow for identifying and validating characteristic genes associated with immunonephropathy. The schematic overview illustrates the multi-step bioinformatic and experimental workflow used in this study. Transcriptomic data from patients with ANCA-associated nephritis, membranous nephropathy (MN), minimal change disease (MCD), and focal segmental glomerulosclerosis (FSGS) were obtained from the GEO database. Plasma membrane homeostasis–related genes were extracted and subjected to differential expression analysis, followed by functional enrichment through Gene Ontology (GO) and Kyoto Encyclopedia of Genes and Genomes (KEGG) analyses. Candidate characteristic genes were screened using least absolute shrinkage and selection operator (LASSO) regression and support vector machine–recursive feature elimination (SVM-RFE) algorithms. Overlapping genes identified by both methods were defined through Venn diagram analysis. The diagnostic efficacy of these genes was evaluated by receiver operating characteristic (ROC) analysis, followed by gene set enrichment analysis (GSEA) and immune infiltration correlation in ANCA, MN, MCD, and FSGS samples. Finally, experimental validation was performed using quantitative real-time PCR (qRT-PCR) and immunofluorescence to confirm the bioinformatic findings.

The overall workflow of this study is illustrated in **Figure 1**. Gene expression profiles of glomeruli from immune nephropathy and living donor kidney biopsy samples were retrieved from the Gene Expression Omnibus (GEO, https://www.ncbi.nlm.nih.gov/geo/) database. Specifically, dataset GSE108109 (platform: GPL22945, Affymetrix Human Genome U133 Plus 2.0 Array) was selected, including a total of 111 samples, of which 105 were immune nephropathy samples and 6 were healthy glomerular controls. Among the immune nephropathy samples, 30 were diagnosed with FSGS, 16 with MCD, 44 with MN, and 15 with AAV (14). Raw CEL files were downloaded and preprocessed using the Robust Multi-array Average (RMA) algorithm for background correction, normalization, and summarization through the “affy” package (v1.78.0) in R software (v4.3.1). Probe sets were mapped to official gene symbols according to the latest HGNC database. In addition, a total of 178 plasma membrane homeostasis-related genes (PMHGs) were retrieved from the GeneCards database (https://www.genecards.org/). Candidate genes were identified by selecting the top 5% of genes based on the combined relevance scores associated with necroptosis, pyroptosis, and ferroptosis. The complete list of PMHGs is provided in **Supplementary Table 1**.

### 2.2 Differentially expressed genes identification

Normalized expression matrices were log2-transformed before analysis. The expression profiles of 178 PMHGs were extracted from the GSE108109 dataset, and differentially expressed PMHGs (DE-PMHGs) between immune nephropathy and healthy glomerular tissues were identified using the Wilcoxon rank-sum test in R. Genes with an adjusted p-value < 0.05 and an absolute log2 fold change ≥ 1 were considered statistically significant. Multiple testing correction was performed using the Benjamini–Hochberg (BH) method to control the false discovery rate (FDR). Visualization of DE-PMHGs was conducted by volcano plots and hierarchical clustering heatmaps generated with the “ggplot2” and “pheatmap” packages (v1.0.12).

### 2.3 Functional annotation

To investigate the potential biological functions of the DE-PMHGs, Gene Ontology (GO) and Kyoto Encyclopedia of Genes and Genomes (KEGG) enrichment analyses were performed using the “clusterProfiler” package (v4.4.4) in R. The background gene set was defined as all genes detected in the GSE108109 dataset. Significantly enriched GO terms, including biological processes (BP), molecular functions (MF), and cellular components (CC), as well as KEGG pathways, were identified based on an adjusted p-value < 0.05. The enrichment results were visualized using the “enrichplot” and “ggplot2” packages to display the top-ranked pathways and functional annotations relevant to immune nephropathy.

### 2.4 Best gene biomarkers for the diagnosis of immunonephropathy

To identify the most robust diagnostic biomarkers, we implemented a stability selection framework for both LASSO and SVM-RFE algorithms. Instead of a single model run, we performed 100 iterations of stratified train/test splitting, with 80% of the data used for training and 20% reserved for evaluating feature stability in each iteration. Only genes that achieved a selection frequency ≥ 60% across these 100 iterations were retained as finalized characteristic genes. To rigorously evaluate the diagnostic reliability and prevent overfitting, we further conducted 5,000 independent stratified splits. In each split, the model direction was determined on the training set, and the Area Under the Curve (AUC) was calculated on a held-out test set (20% of samples) that the model had never encountered during training, providing a distribution of predictive accuracy on unseen data (15). In addition, correlations between the characteristic genes and clinical parameters of kidney diseases were analyzed through the Nephroseq v5 database (http://v5.nephroseq.org/).

### 2.5 Immune infiltration analysis

The xCell algorithm (https://xcell.ucsf.edu/) (16) was employed to estimate the relative abundance of 64 immune and stromal cell types based on gene expression data. Normalized expression profiles from GSE108109 were used as the input, and cell type-specific enrichment scores were generated for each sample. Immune cell types not typically expressed in renal tissues were excluded from downstream analysis. The Wilcoxon rank-sum test was used to compare differences in immune cell infiltration among ANCA, FSGS, MCD, MN, and normal samples, with *p* < 0.05 considered statistically significant. The association between characteristic gene expression and immune infiltration levels was examined using Spearman correlation analysis, and correlation heatmaps were generated using the “corrplot” package.

### 2.6 Animal model generated and quantitative real-time PCR (qRT-PCR) analysis

All animal experiments were conducted in accordance with the “China Guide for the Protection and Use of Laboratory Animals” and were approved by the Laboratory Animal Management and Ethics Committee of Fujian Medical University (Approval No. IACUC FJMU 2023-Y-0873). Male C57BL/6J mice (8–10 weeks old, 20–25 g) were obtained from the Animal Center of Fujian Medical University and housed in a specific pathogen-free (SPF) environment under controlled temperature (22 ± 2 °C), humidity (50 ± 5%), and a 12-hour light/dark cycle with free access to food and water.

To simulate the pathology of ANCA - associated nephritis, we employed an established accelerated anti- myeloperoxidase (MPO) -induced crescentic glomerulonephritis model that combines MPO - specific autoimmunity with anti- glomerular basement membrane (GBM) -mediated glomerular stress, which more consistently reproduces severe crescentic lesions than MPO immunization alone. Male C57BL/6J mice were used to reduce potential variability related to sex hormones and estrous cycling. Specifically, mice were immunized intraperitoneally on day 0 with 20 μg of murine MPO emulsified in Freund’s complete adjuvant (FCA, Sigma-Aldrich, USA) to initiate anti-MPO antibody production. On day 7, mice were boosted subcutaneously with 10 μg MPO in Freund’s incomplete adjuvant (FIA, Sigma-Aldrich). To accelerate the development of necrotizing glomerular injury, anti- GBM nephritis was induced on day 16 by intraperitoneal injection of 20 μg/g body weight of sheep anti-mouse GBM globulin (ProSci, USA) (17, 18).

qRT-PCR was performed using the Taq Pro Universal SYBR qPCR Master Mix (Q712-0, Novogene Bioinformatics Technology Co., Ltd., Nanjing). The reaction mixture (20 µL total volume) contained 10 µL of SYBR qPCR Master Mix, 0.4 µL of each forward and reverse primer, 1 µL of cDNA, and 1 µL of ddH2O. The PCR amplification was conducted under the following conditions: an initial denaturation at 95 °C for 10 seconds, followed by 40 cycles of denaturation at 95 °C for 30 seconds and annealing at 60 °C for 30 seconds. Relative mRNA expression levels were calculated using the 2^-△△CT method. The primers used in the qRT-PCR are listed below: TP53 (F: CTCTCCCCCGCAAAAGAAAAA; R: CGGAACATCTCGAAGCGTTTA), SLC40A1 (F: ACCAAGGCAAGAGATCAAACC; R: AGACACTGCAAAGTGCCACAT), SLC39A7 (F: TGAAAGCATCTGGCATGGG; R: TGGAGGCTATCGTGGGAGTG), KEAP1 (F: TGCCCCTGTGGTCAAAGTG; R: GGTTCGGTTACCGTCCTGC), IPMK (F: GAAGGAAGGCGTCTCCAAGTT; R: CGCCAAAGTTCTATCGTTTGC), TNIP1 (F: CCAGGTACATCCTGCTACCAG; R: CGCCATTGGACGGAGATTTG), NCOA4 (F: GCCCTACAATGTGAGTGATTGG; R: ACTGGTGCAAGGCTCGTTG),SAT1 (F: GAGAACACCCCTTCTACCACT; R: GCCTCTGTAATCACTCATCACGA).

### 2.7 Laser microdissection of frozen kidney sections

Frozen kidney tissue sections from MPO-induced mice were immediately fixed in pre-cooled 75% ethanol for 2 minutes, followed by staining with cresyl violet for 1–2 minutes with gentle agitation. The stained sections were sequentially dehydrated in 75%, 90%, and 100% ethanol for 5 seconds each and then fixed in 100% ethanol for 1 minute. After air-drying, glomerular structures were microdissected using a Leica LMD7000 confocal laser microdissection system (Leica Microsystems, Germany). All microdissected glomeruli were collected into microtubes containing TRIzol reagent and stored at –20 °C until RNA extraction.

### 2.8 RNA extraction from laser-microdissected frozen samples

Total RNA was extracted from the laser-microdissected glomerular samples using the RNeasy Micro Kit (Qiagen, Germany) following the manufacturer’s protocol. Briefly, tissues were lysed in buffer RL supplemented with 1% β-mercaptoethanol, and carrier RNA was added when the total cell number was below 5,000. After homogenization and ethanol addition, lysates were transferred to purification columns. Sequential washes with RW1 and RW buffers were performed, followed by on-column DNase I treatment to remove genomic DNA. RNA was eluted in RNase-free water and quantified using a NanoDrop 2000 spectrophotometer (Thermo Fisher Scientific, USA).

### 2.9 Immunofluorescence

Cryosections (4 μm) of renal tissue were fixed in ice-cold acetone for 15 minutes, washed with phosphate-buffered saline (PBS), and incubated with primary antibodies overnight at 4 °C. The following primary antibodies were used: anti-p53 (Abcam, ab32389, 1:200) and anti-TNIP1 (Proteintech, 11624-1-AP, 1:100). After washing, sections were incubated with Alexa Fluor^®^ 488 and Alexa Fluor^®^ 594-conjugated secondary antibodies (Abcam) for 1 hour at room temperature. Nuclei were counterstained with DAPI (D3571, Invitrogen). Images were captured using an Olympus FV3000 confocal microscope (Olympus, Japan). Quantitative analysis was performed in a blinded manner using three randomly selected fields per section, and the number of positive cells was quantified with ImageJ software (v1.54).

### 2.10 Statistical analysis

All statistical analyses were conducted using R software (v4.3.1). Two-group comparisons were performed using the Wilcoxon rank-sum test. Correlation analyses between DE-PMHGs and clinical features or immune infiltration levels were assessed by Pearson or Spearman correlation, depending on data distribution. Venn diagrams were plotted using the “Jvenn” package, and all results were visualized using R’s base plotting and ggplot2 functions. All p-values were two-tailed, with p < 0.05 considered statistically significant. Data are expressed as mean ± standard error of the mean (SEM) unless otherwise specified.

## 3 Results

### 3.1 Differential gene expression and functional enrichment in immunonephropathy

**Figure 2:**
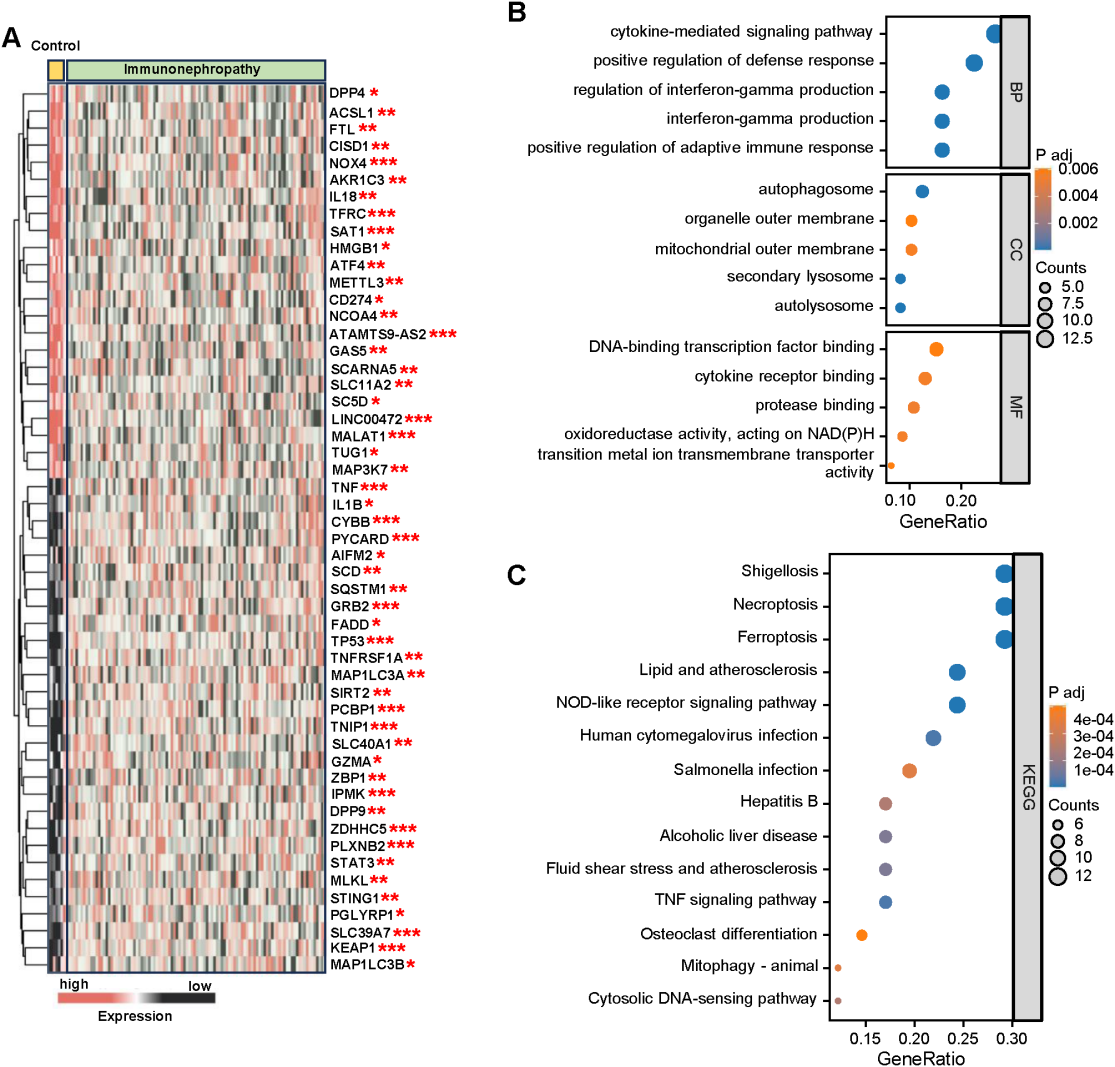
Differential expression analysis and functional enrichment of genes in immunonephropathy. **(A)** Heatmap illustrating the expression profiles of plasma membrane homeostasis–related genes in immunonephropathy versus control samples. The color scale represents relative expression levels, with red indicating upregulation and blue indicating downregulation. Statistical significance levels are denoted as *p* < 0.05 (*), *p* < 0.01 (**), and *p* < 0.001 (***). **(B)** Gene Ontology **(GO)** enrichment analysis showing the top significantly enriched biological processes (BP), cellular components (CC), and molecular functions (MF) of the differentially expressed genes. Dot size indicates the number of genes enriched in each term, and color intensity reflects the adjusted p-value. Enriched terms such as cytokine-mediated signaling and immune response regulation highlight the contribution of immune-related pathways to disease pathogenesis. **(C)** Kyoto Encyclopedia of Genes and Genomes (KEGG) pathway enrichment analysis identifying key signaling pathways associated with immunonephropathy. Dot size corresponds to the number of genes enriched, and color indicates adjusted p-values. Significantly enriched pathways—including necroptosis, NOD-like receptor signaling, and Shigellosis—suggest that regulated cell death and innate immune activation are central to the molecular mechanisms underlying immunonephropathy.

To explore the molecular alterations associated with immunonephropathy, we performed differential expression analysis of genes in immunonephropathy samples compared to control samples. A total of 52 differentially expressed genes (DEGs) were identified from 149 genes in immunonephropathy versus control samples in the dataset. Among them, 29 genes were upregulated, while 23 genes showed downregulation (**Figure 2A**). To further investigate the functional relevance of these differentially expressed genes, we conducted GO enrichment analysis (**Figure 2B**). The analysis revealed that several BP, such as cytokine-mediated signaling, positive regulation of immune responses, and interferon-gamma production, were significantly enriched. These findings suggest a central role of immune-related processes in the pathogenesis of immunonephropathy. Additionally, the analysis identified enrichment in CC such as the autophagosome and mitochondrial outer membrane, as well as MF like DNA-binding transcription factor binding and protease binding, underscoring the complex molecular machinery involved in immunonephropathy. We further performed KEGG pathway enrichment analysis to identify the key signaling pathways associated with the differentially expressed genes (**Figure 2C**). Several immune-related pathways, including Shigellosis, Necroptosis, and NOD-like receptor signaling, were significantly enriched, highlighting their potential involvement in immune dysregulation and inflammatory responses in immunonephropathy. In addition, pathways related to lipid metabolism, atherosclerosis, and TNF signaling were also identified, providing further insight into the systemic nature of the disease. These findings suggest that the dysregulation of immune and metabolic pathways plays a crucial role in the development and progression of immunonephropathy, offering potential targets for therapeutic intervention.

### 3.2 Screening and performance evaluation of characteristic genes using LASSO and SVM Models

**Figure 3:**
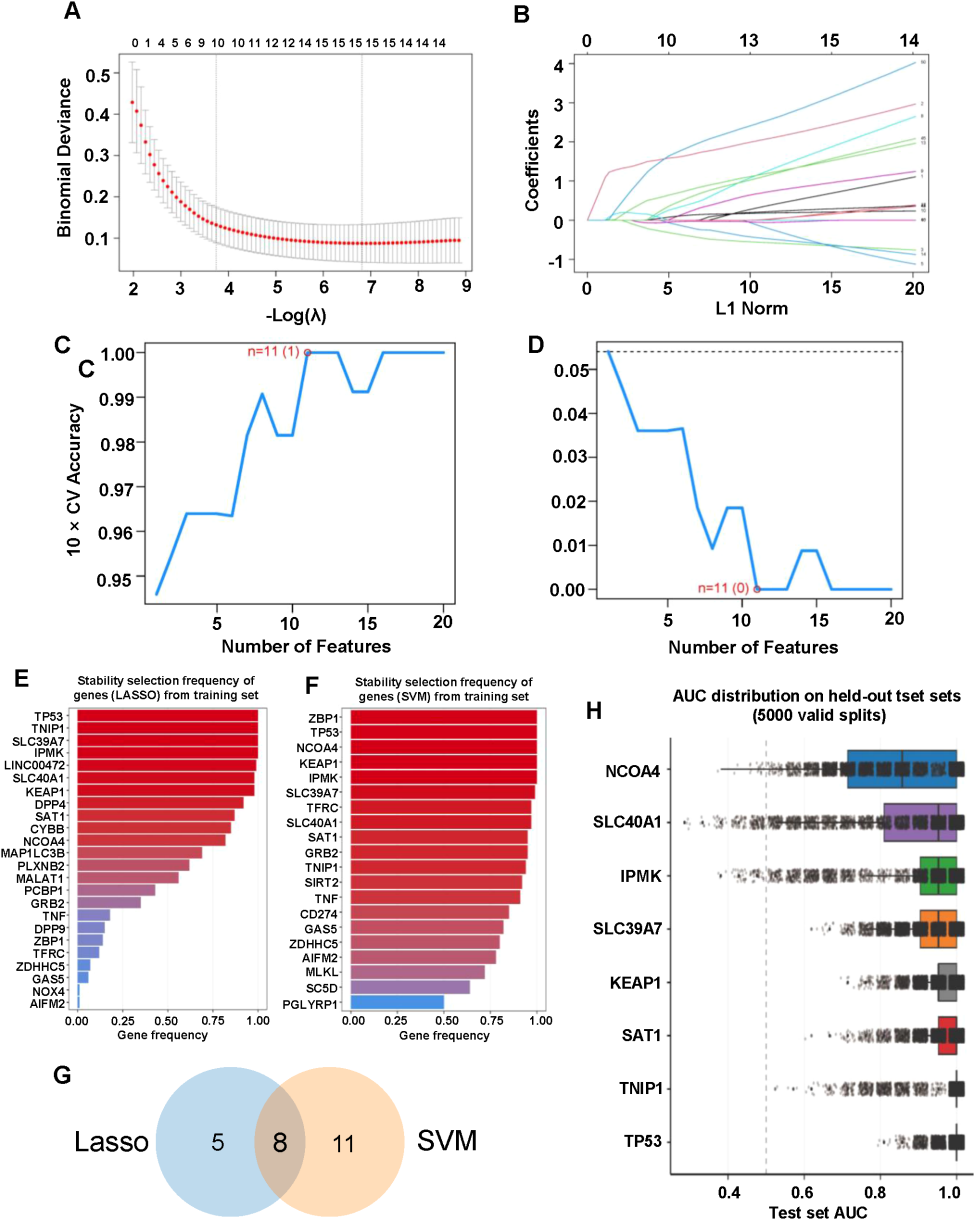
Screening and performance evaluation of characteristic genes using LASSO and SVM models. **(A)** Ten-fold cross-validation curve of the LASSO logistic regression model showing binomial deviance versus −log(λ). Red dots represent the mean deviance at each λ, and gray bars indicate ±1 standard error. Vertical dashed lines denote the optimal λ values, and the numbers above the plot indicate the number of nonzero coefficients retained at each λ. **(B)** LASSO coefficient profiles for candidate genes plotted against the L1 norm, illustrating the shrinkage of coefficients toward zero as penalization increases. **(C, D)** Performance of the SVM-RFE model evaluated by cross-validation across different feature set sizes. The cross-validation accuracy **(C)** and classification error **(D)** are shown as functions of the number of selected features, with the optimal model achieving the best performance when 11 genes are selected (n = 11). **(E)** Stability selection frequency of genes in the LASSO model across 100 stratified training resamples (80% training/20% testing per iteration). Genes with a selection frequency ≥60% were retained as stable candidates. **(F)** Stability selection frequency of genes in the SVM-RFE model across 100 stratified training resamples, with genes meeting the ≥60% selection threshold retained. **(G)** Venn diagram illustrating the overlap between the stable genes identified by LASSO and SVM-RFE. Eight shared genes (*IPMK*, *TP53*, *SLC40A1*, *NCOA4*, *SLC39A7*, *KEAP1*, *TNIP1*, and *SAT1*) were defined as the final characteristic gene set. **(H)** AUC distribution of each characteristic gene evaluated on held-out test sets across 5,000 independent stratified splits (20% test sets not seen during training). Each point represents one split, and boxplots summarize the overall performance distribution for each gene.

To identify key genes associated with immunonephropathy, we employed an integrated feature selection strategy combining LASSO and SVM models1. In the LASSO model, the relationship between the regularization parameter (lambda) and the binomial deviance was explored via cross-validation to minimize deviance and optimize predictive performance (**Figure 3A**). The coefficient paths for each feature were analyzed as a function of the L1 norm, illustrating the progressive exclusion of less significant features (**Figure 3B**). To ensure the robustness of the selected genes, we performed 100 iterations of stratified train/test splitting to calculate the stability selection frequency for each gene, retaining those that consistently appeared across different data subsets (**Figure 3E**). The SVM-RFE algorithm was similarly implemented to iteratively select genes with the highest contribution to classification accuracy. The model’s performance was evaluated through 10-fold cross-validation accuracy and error curves, with the optimal feature set identified at the point of maximum accuracy (**Figures 3C, D**). Stability selection for the SVM-RFE model further confirmed the most reliable features across 100 iterations (**Figure 3F**). By intersecting the robust genes identified by both the LASSO and SVM models through Venn diagram analysis, we identified eight core characteristic genes: *IPMK*, *TP53*, *SLC40A1*, *NCOA4*, *SLC39A7*, *KEAP1*, *TNIP1*, and *SAT1* (**Figure 3G**). The diagnostic performance of these eight genes was rigorously validated on unseen data through 5,000 independent stratified splits. In each split, the model direction was determined on the training set, and the AUC was calculated on a held-out test set (20% of samples). The resulting AUC distributions demonstrated excellent and stable predictive power for all eight genes (**Figure 3H**). Specifically, *TP53* and *TNIP1* exhibited the highest diagnostic accuracy, with median AUC values of 1.000 (IQR: [1.000, 1.000] for *TP53* and [1.000, 1.000] for *TNIP1*) and mean AUCs of 0.987 ± 0.029 and 0.970 ± 0.074, respectively. Other genes also maintained high performance, including *SAT1* (Median: 1.000, Mean: 0.963 ± 0.055), *KEAP1* (Median: 0.952, Mean: 0.958 ± 0.051), *SLC39A7* (Median: 0.952, Mean: 0.929 ± 0.072), *IPMK* (Median: 0.952, Mean: 0.913 ± 0.134), *SLC40A1* (Median: 0.952, Mean: 0.883 ± 0.162), and *NCOA4* (Median: 0.857, Mean: 0.847 ± 0.139). These findings underscore the high discriminatory capability and cross-validation stability of the identified eight-gene signature as potential biomarkers for immunonephropathy.

### 3.3 Expression and functional enrichment of characteristic genes in immunonephropathy

**Figure 4:**
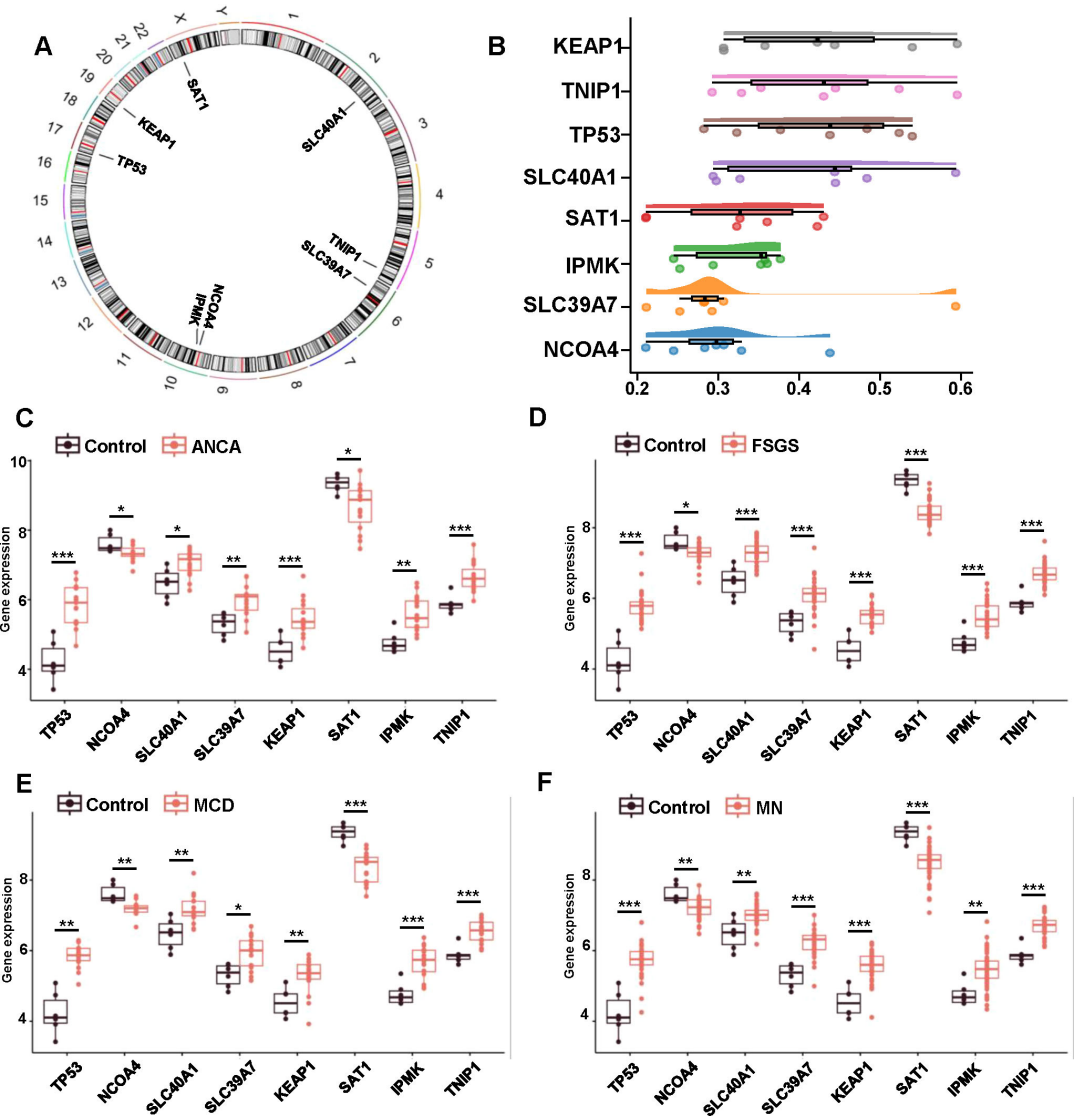
Expression analysis and functional enrichment of characteristic genes in immunonephropathy. **(A)** Circular plot depicting the chromosomal distribution of the eight characteristic genes (*KEAP1, IPMK, SAT1, TP53, TNIP1, SLC40A1, SLC39A7*, and *NCOA4*) across the human genome. **(B)** Functional similarity analysis of the characteristic genes using the “GOSemSim” package based on Gene Ontology (GO) terms. The heatmap reflects semantic similarity scores, with higher scores indicating closer functional relationships and shared biological roles among genes. **(C–F)** Box plots comparing expression levels of characteristic genes in different immune-mediated nephropathies—**(C)** ANCA, **(D)** FSGS, **(E)** MCD, and **(F)** MN—relative to control samples. Red boxes indicate upregulation and blue boxes indicate downregulation. Statistical significance levels are denoted as p < 0.05 (*), p < 0.01 (**), and p < 0.001 (***).

**Figure 5:**
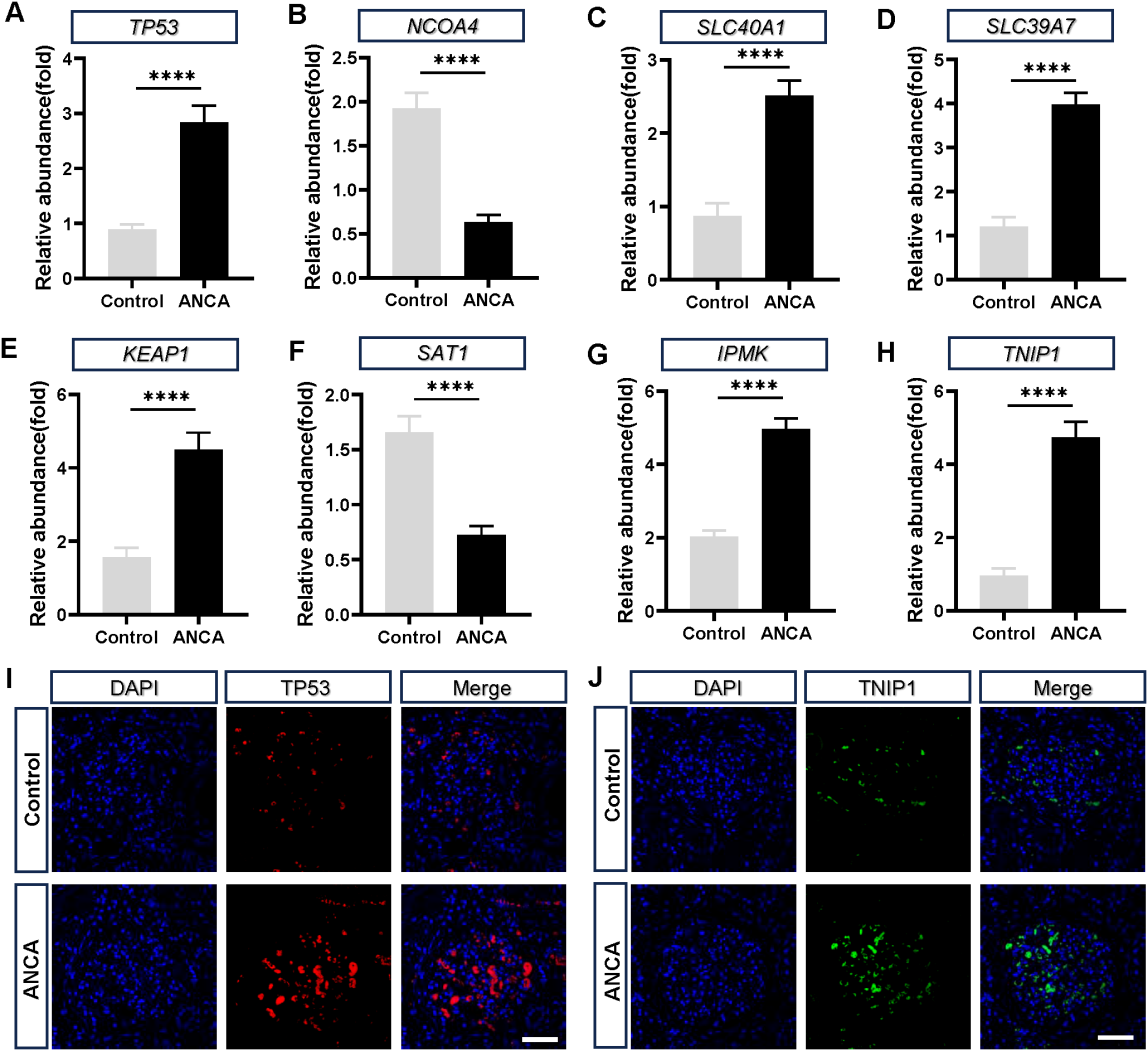
Expression analysis of characteristic genes in ANCA-associated nephritis. **(A–H)** Quantitative real-time PCR (qRT-PCR) analysis of relative gene expression in laser-microdissected glomeruli isolated from kidneys of ANCA-induced nephritis mice and control mice. *TP53* **(A)**, *SLC40A1* **(C)**, *SLC39A7* **(D)**, *KEAP1* **(E)**, *IPMK* **(G)**, and *TNIP1* **(H)** are significantly upregulated in the ANCA group, whereas *NCOA4* **(B)** and *SAT1* **(F)** are markedly downregulated compared with controls. Data are expressed as mean ± SEM; *p* < 0.0001(****). **(I, J)** Immunofluorescence staining of laser-microdissected glomeruli from ANCA nephritis mouse kidneys for TP53 **(I)** and TNIP1 **(J)** The control and ANCA groups were stained with DAPI **(blue)** and either anti-TP53 (red) or anti-TNIP1 (green). Co-localization of TP53 and TNIP1 with the glomeruli indicates consistent upregulation in ANCA-associated nephritis, supporting the qPCR data. Scale bar = 20 µm.

We first examined the chromosomal locations of the characteristic genes identified in the study, including *KEAP1*, *SAT1*, *TP53*, *TNIP1*, *SLC40A1*, *SLC39A7*, and *NCOA4*, which are distributed across multiple chromosomes (**Figure 4A**). GO enrichment analysis using the GOSemSim package (**Figure 4B**) revealed significant functional similarities among these genes. We further assessed the expression of these genes in different types of immunonephropathy using box plots (**Figures 4C–F**). Compared with the control group, consistent and significant changes in gene expression were observed in the disease groups (ANCA, FSGS, MCD and MN). Furthermore, we established a mouse model of ANCA-associated nephritis by intraperitoneal injection of MPO and performed qPCR on laser-microdissected glomeruli to validate the accuracy of the characteristic gene expression (**Figures 5A–H**). Additionally, we performed immunofluorescence staining on laser-microdissected glomeruli from kidney samples of mice with ANCA-associated nephritis to validate the diagnostic potential of the two most diagnostically significant protein, TP53 and TNIP1, and obtained results consistent with the qPCR findings (**Figures 5I, J**).

**Figure 6:**
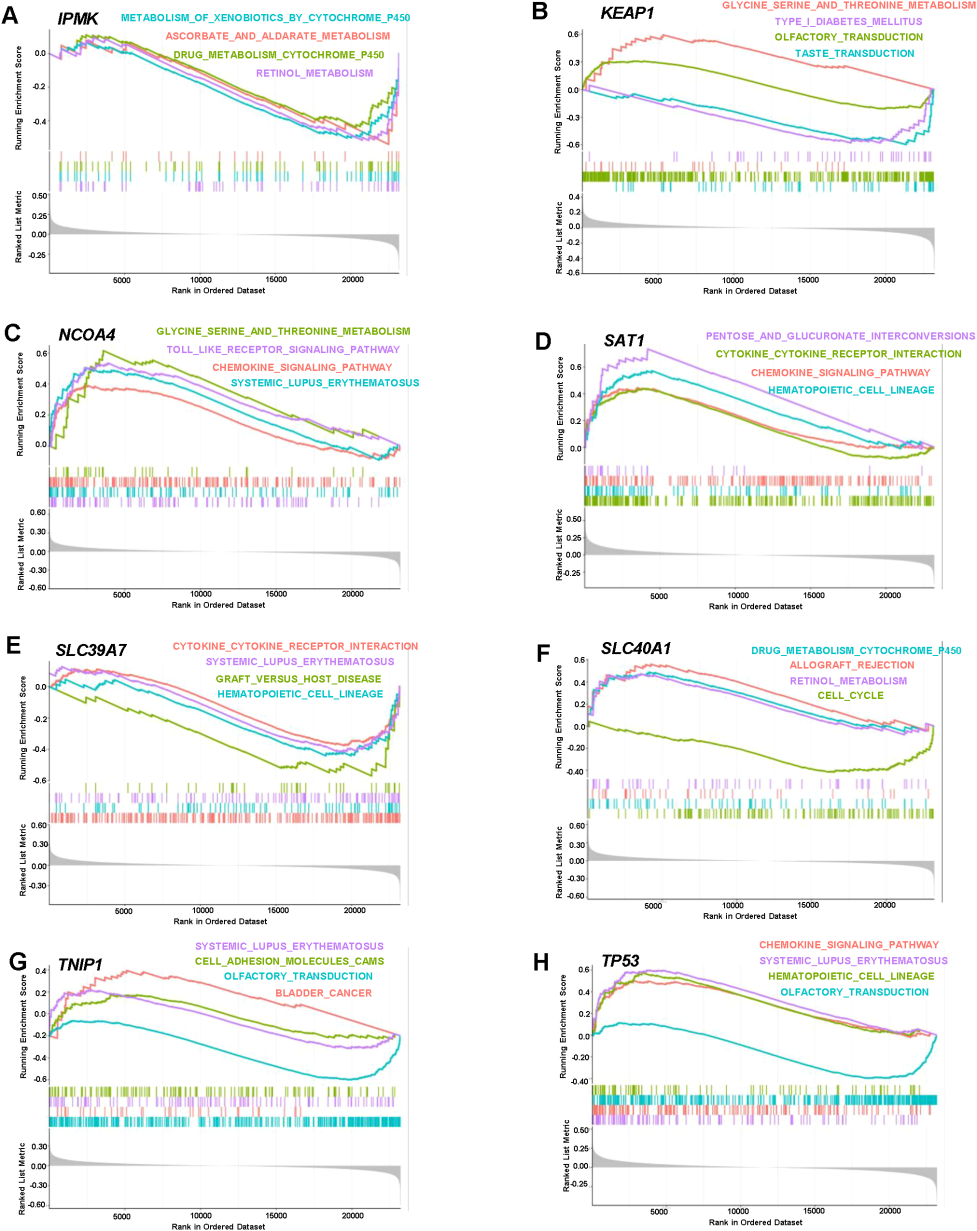
Gene set enrichment analysis (GSEA) of characteristic genes in immunonephropathy. GSEA was performed to assess the biological pathways enriched for each characteristic gene within the ranked transcriptomic dataset. The x-axis represents the gene rank across the ordered list, while the y-axis denotes the enrichment score (ES). Vertical bars indicate the positions of genes contributing to the core enrichment for each pathway. **(A)** IPMK shows significant enrichment in xenobiotic metabolism, retinol metabolism, and cytochrome P450–related drug metabolism pathways. **(B)** KEAP1 is enriched in glycine, serine, and threonine metabolism, taste transduction, and diabetes-associated pathways. **(C)** NCOA4 displays enrichment in glycine and threonine metabolism and systemic lupus erythematosus (SLE) pathways. **(D)** SAT1 is involved in cytokine signaling, hematopoietic cell lineage, and glucuronide interconversion pathways. **(E)** SLC39A7 shows enrichment in immune response–related pathways, including cytokine receptor interaction and SLE. **(F)** SLC40A1 is enriched in drug metabolism, allograft rejection, and cell cycle regulation pathways. **(G)** TNIP1 demonstrates enrichment in SLE, cell adhesion, and bladder cancer–associated signaling. **(H)** TP53 is strongly enriched in chemokine signaling, SLE, and hematopoietic cell lineage pathways.

Furthermore, we performed Gene Set Enrichment Analysis (GSEA) to further explore the biological relevance of the characteristic genes. *IPMK* (**Figure 6A**) was significantly enriched in pathways such as xenobiotic metabolism and drug metabolism, while *KEAP1* (**Figure 6B**) was enriched in glycine, serine, and threonine metabolism and diabetes-related pathways. *NCOA4* (**Figure 6C**) showed strong enrichment in pathways involved in systemic lupus erythematosus and glycine, serine, and threonine metabolism, suggesting its role in immune responses. *SAT1* (**Figure 6D**) was linked to pathways such as cytokine signaling and hematopoietic cell lineage. *SLC39A7* (**Figure 6E**) exhibited enrichment in immune response pathways, including cytokine receptor interaction and systemic lupus erythematosus, while *SLC40A1* (**Figure 6F**) was enriched in drug metabolism, allograft rejection, and cell cycle regulation. *TNIP1* (**Figure 6G**) was enriched in pathways related to systemic lupus erythematosus and cell adhesion, and *TP53* (**Figure 6H**) was significantly enriched in pathways such as chemokine signaling, systemic lupus erythematosus, and hematopoietic cell lineage. These findings underscore the involvement of the characteristic genes in immune and metabolic pathways, providing valuable insights into the molecular mechanisms of immunonephropathy and identifying potential therapeutic targets for future research.

### 3.4 Immune cell profiling and correlation with characteristic genes in immunonephropathy

**Figure 7:**
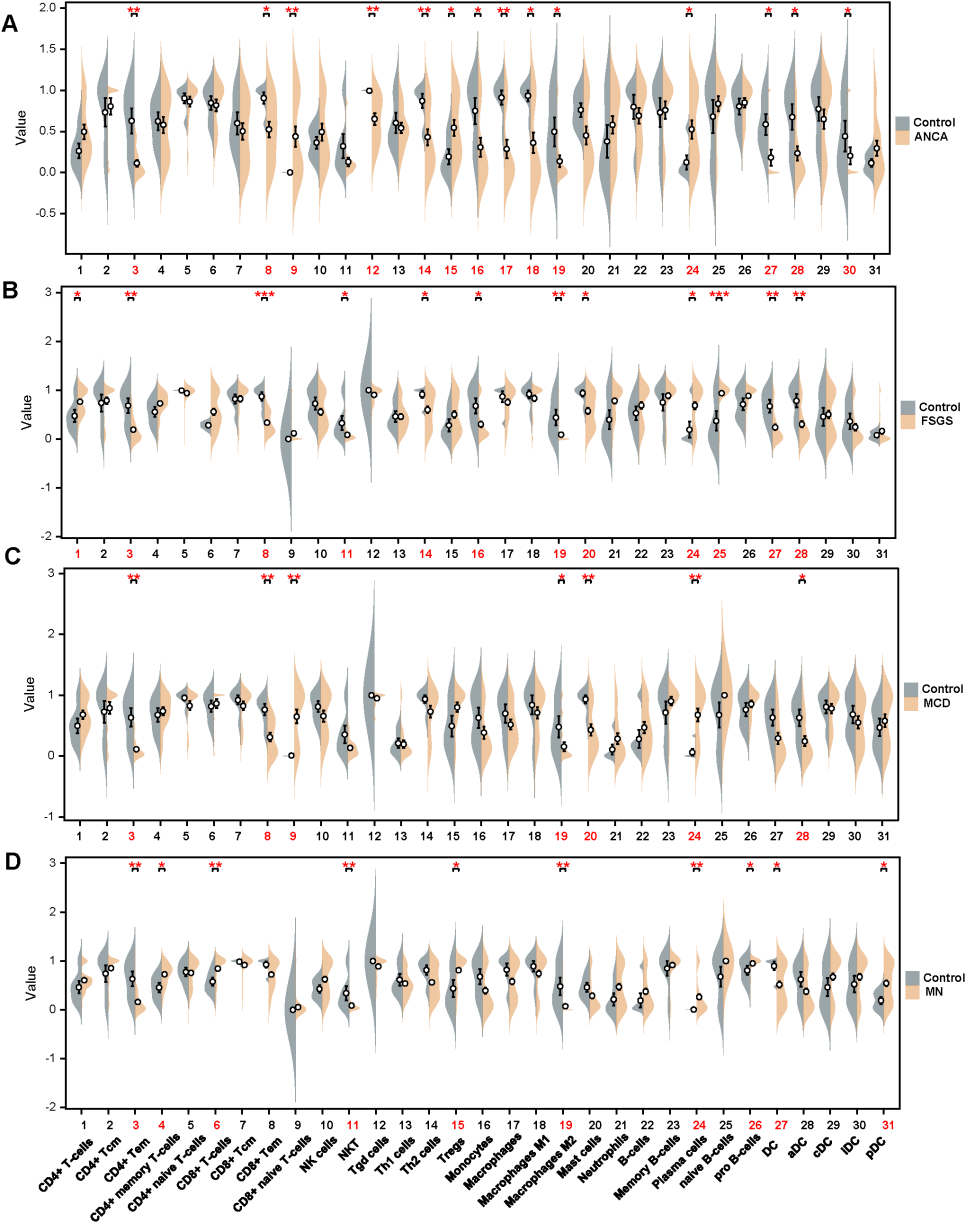
Immune cell composition analysis in different types of immunonephropathy. **(A–D)** Violin plots showing the relative abundance of immune cell types in patients with different forms of immunonephropathy compared with healthy controls. Each panel represents a distinct condition: **(A)** ANCA-associated nephritis, **(B)** focal segmental glomerulosclerosis (FSGS), **(C)** minimal change disease (MCD), and **(D)** membranous nephropathy (MN). The x-axis denotes immune cell subsets, and the y-axis indicates normalized infiltration scores. Statistical significance is denoted as *p* < 0.05 (*), *p* < 0.01 (**), and *p* < 0.001 (***).

Using the xCell algorithm, we compared the distribution of immune cell subsets across different types of immunonephropathy (ANCA, FSGS, MCD, and MN). In the ANCA group (**Figure 7A**), significant increases were observed in CD8^+^ naive T cells and regulatory T cells (Tregs), while CD4^+^ Tem, CD8^+^ Tem, Tγδ cells, Th2 cells, monocytes, macrophages (including M1 and M2), dendritic cells (DCs), activated DCs (aDCs), and immature DCs (iDCs) were significantly reduced, indicating suppression of effector and innate immune components with a compensatory regulatory T cell expansion. FSGS patients exhibited elevated levels of CD4^+^ T cells, plasma cells, and naive B cells, alongside a significant reduction in CD4^+^ Tem, CD8^+^ Tem, NKT cells, Th2 cells, monocytes, macrophage M2, mast cells, DCs, and aDCs, suggesting a shift toward lymphocyte-driven immunity and a concomitant loss of innate immune regulation (**Figure 7B**). In the MCD cohort, CD8^+^ naive T cells and plasma cells were significantly increased, while CD4^+^ Tem, macrophage M2, mast cells, and aDCs were decreased (**Figure 7C**). These findings reflect a profile of enhanced naive and humoral activation with suppression of innate immune effectors. In contrast, MN patients showed increased proportions of CD4^+^ memory T cells, CD8^+^ T cells, Tregs, plasma cells, pro-B cells, and plasmacytoid DCs (pDCs), whereas NKT cells, macrophage M2, and DCs were significantly decreased. This pattern suggests concurrent activation of both adaptive effector and regulatory arms, along with impairment in regulatory innate subsets (**Figure 7D**). Collectively, these results highlight the heterogeneity of immune dysregulation across glomerular diseases, with each entity exhibiting a unique immune signature involving distinct combinations of adaptive and innate immune alterations.

**Figure 8:**
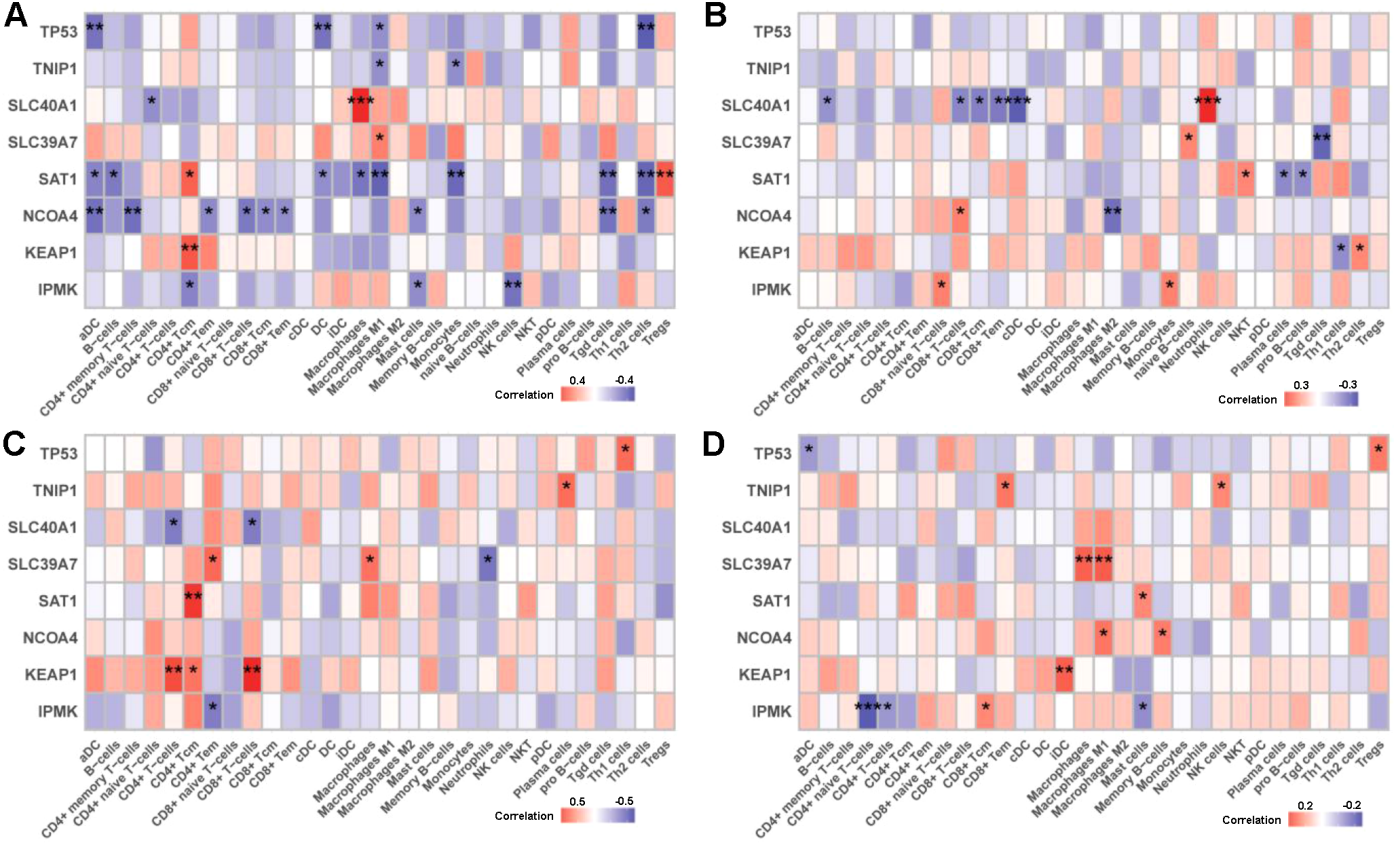
Correlation between characteristic genes and immune cell subsets in different types of immunonephropathy. Heatmaps illustrating the correlation between the expression levels of characteristic genes (*KEAP1*, *IPMK*, *SAT1*, *TP53*, *TNIP1*, *SLC40A1*, *SLC39A7*, and *NCOA4*) and the relative abundance of immune cell subsets across four disease types: **(A)** ANCA, **(B)** FSGS, **(C)** MCD, and **(D)** MN. Each row represents an immune cell type, and each column corresponds to a characteristic gene. The color gradient reflects correlation strength and direction, with red indicating a positive correlation and blue indicating a negative correlation. Statistical significance levels are denoted as *p* < 0.05 (*), *p* < 0.01 (**), and *p* < 0.001 (***).

Correlation analysis was performed to investigate the relationship between characteristic genes and immune cell subsets across different immunonephropathy types. In ANCA-associated nephritis, *TP53*, *TNIP1*, and *SAT1* were broadly negatively correlated with antigen-presenting cells (aDCs, DCs) and M1 macrophages, suggesting a potential role in dampening pro-inflammatory responses. Notably, *SAT1* also exhibited a positive correlation with regulatory T cells (Tregs), indicating a possible immunosuppressive function (**Figure 8A**). In FSGS, *SLC40A1* was positively correlated with neutrophils and negatively with multiple lymphocyte subsets, including CD8^+^ T cells and B cells, pointing to a shift toward innate immunity. *NCOA4* displayed a dual immunoregulatory role, enhancing CD8^+^ T and Th2 cell presence while suppressing M2 macrophages and Th1 cells, implying involvement in adaptive immune modulation (**Figure 8B**). In MCD, *KEAP1* was positively associated with multiple T cell subsets, especially CD4^+^ and CD8^+^ cells, indicating possible involvement in T cell activation. Conversely, *SLC40A1* and *IPMK* were negatively correlated with T cells, suggesting gene-specific suppression of adaptive immunity (**Figure 8C**). In MN, *TP53* and *TNIP1* showed positive associations with immunoregulatory cells, including Tregs and NK cells. *IPMK* demonstrated a complex profile, promoting CD8^+^ Tcm cells while inhibiting CD4^+^ subsets and mast cells, indicating selective modulation of T cell compartments (**Figure 8D**). These findings suggest that the same gene may exert distinct immunomodulatory effects across different glomerular diseases. The diverse immune gene signatures highlight potential diagnostic markers or therapeutic targets tailored to specific immune microenvironments.

### 3.5 Correlation between characteristic gene expression and clinical parameters

**Figure 9:**
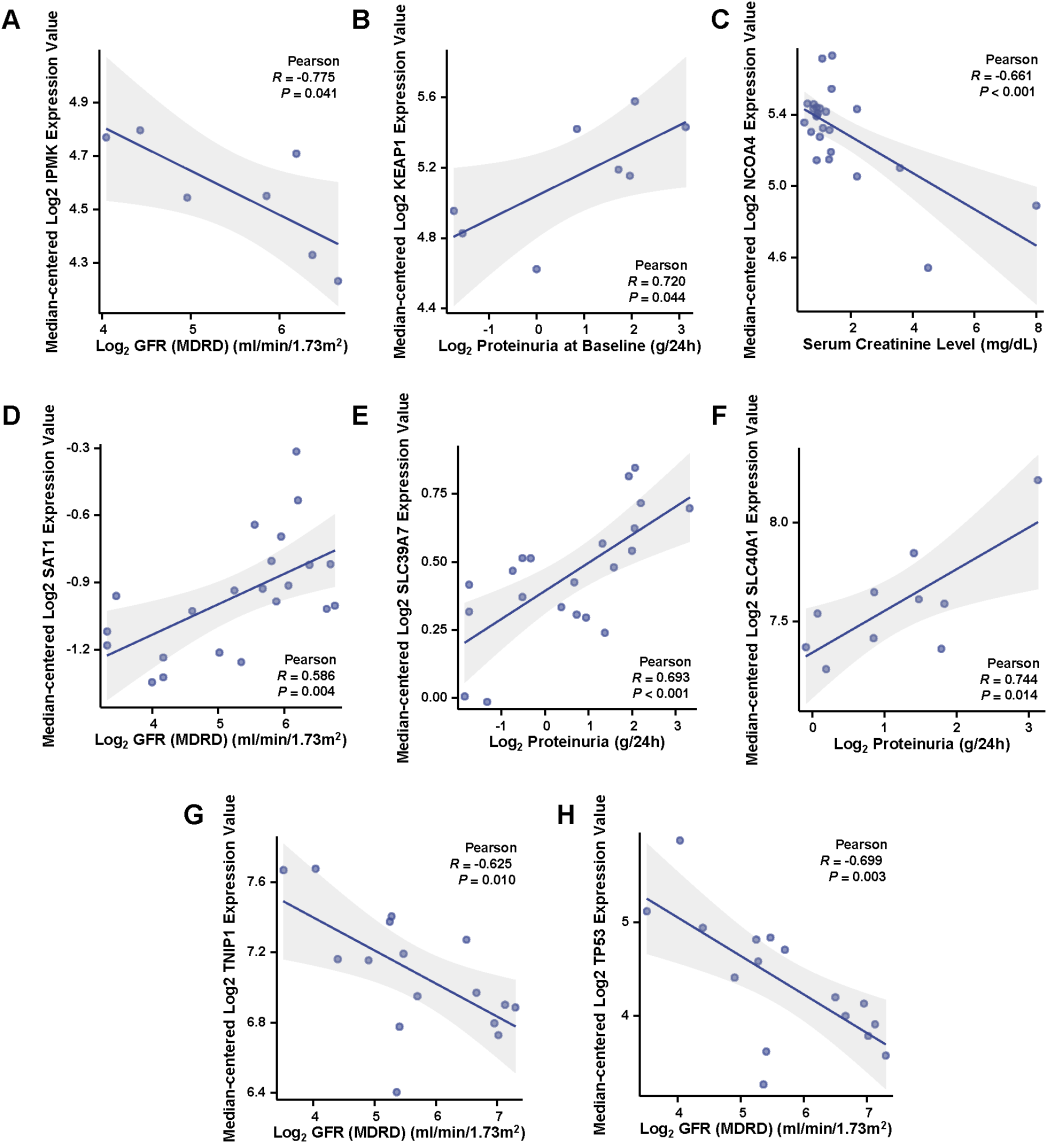
Correlation between characteristic gene expression and clinical parameters. **(A–H)** Scatter plots showing the associations between characteristic gene expression levels and clinical parameters, including estimated glomerular filtration rate (GFR), proteinuria, and serum creatinine. Pearson’s correlation coefficient **(r)** and corresponding p-values are displayed in each panel. **(A)** IPMK expression negatively correlates with GFR (r = –0.775, *p* = 0.041). **(B)** KEAP1 expression positively correlates with proteinuria (r = 0.720, *p* = 0.044). **(C)** NCOA4 expression negatively correlates with serum creatinine (r = –0.661, *p* < 0.001). **(D)** SAT1 expression positively correlates with GFR (r = 0.586, *p* = 0.004). **(E)** SLC39A7 expression positively correlates with proteinuria (r = 0.693, *p* < 0.001). **(F)** SLC40A1 expression positively correlates with proteinuria (r = 0.744, *p* = 0.014). **(G)** TNIP1 expression negatively correlates with GFR (r = –0.625, *p* = 0.010). **(H)** TP53 expression negatively correlates with GFR (r = –0.699, *p* = 0.003).

We examined the correlation between the expression of characteristic genes and key clinical parameters. *IPMK* expression was negatively correlated with GFR (*R* = -0.775, *p* = 0.041), indicating that lower kidney function is associated with increased expression of IPMK (**Figure 9A**). *KEAP1* expression, on the other hand, showed a positive correlation with proteinuria at baseline (*R* = 0.720, *p* = 0.044), suggesting that higher expression of *KEAP1* is linked to elevated proteinuria levels (**Figure 9B**). *NCOA4* expression was negatively correlated with serum creatinine levels (*R* = -0.661, *p* < 0.001), implying that higher *NCOA4* expression is associated with better kidney function (**Figure 9C**). In contrast, *SAT1* expression exhibited a positive correlation with GFR (*R* = 0.586, *p* = 0.004), indicating that higher *SAT1* expression is associated with improved kidney function (**Figure 9D**). A positive correlation between *SLC39A7* expression and proteinuria at baseline was observed (*R* = 0.693, *p* < 0.001), further supporting the role of *SLC39A7* in kidney injury and protein leakage (**Figure 9E**). Similarly, *SLC40A1* expression showed a positive correlation with proteinuria at baseline (*R* = 0.744, *p* = 0.014), suggesting its involvement in protein filtration (**Figure 9F**). Both *TNIP1* (*R* = -0.625, *p* = 0.010) and *TP53* (*R* = -0.699, *p* = 0.003) were negatively correlated with GFR (**Figures 9G, H**). These findings indicate that increased expression of *TNIP1* and *TP53* is associated with decreased kidney function. These results demonstrate significant correlations between the expression of characteristic genes and clinical parameters such as GFR, proteinuria, and serum creatinine levels. These correlations suggest that these genes may serve as potential biomarkers for assessing kidney function and disease progression in immunonephropathy.

## 4 Discussion

This study explores the molecular landscape of diverse immune-mediated glomerular diseases. While these conditions possess distinct immunopathogenic origins—ranging from systemic autoimmune-mediated inflammation in AAV to complement-mediated injury in MN and primary podocyte stress in FSGS and MCD—our findings suggest that they may share a convergent downstream mechanistic pathway. Specifically, dysregulated cytoplasmic membrane homeostasis acts as a potential bridge connecting these varied immune triggers to regulated cell death and progressive glomerular damage (19). Within our proposed hypothetical model, we suggest that once disrupted, membrane instability may potentially trigger the release of DAMPs, thereby amplifying inflammatory signaling and accelerating glomerular injury (20). Our integrated transcriptomic and immune-infiltration analyses support a potential link between molecular dysfunction and immune-mediated damage, suggesting a conceptual framework where necroptosis, pyroptosis, and ferroptosis may collectively contribute to membrane destabilization across distinct pathological subtypes (21).

We identified eight core genes—*IPMK*, *TP53*, *SLC40A1*, *NCOA4*, *SLC39A7*, *KEAP1*, *TNIP1*, and *SAT1*— that are potentially involved in oxidative defense, iron metabolism, immune signaling, and cell-death pathways in glomerular cells. Rather than viewing the identified eight-gene signature as a mere list of biomarkers, we propose that these genes constitute an integrated, multi-layered resilience axis that characterizes glomerular cell survival and susceptibility to injury. Within this conceptual framework, an Ion-dependent sensitization module is formed by the synergy between iron metabolism markers (*SLC40A1* and *NCOA4*) and the zinc transporter (*SLC39A7*), which appears to modulate the fundamental susceptibility of the plasma membrane to lipid peroxidation; specifically, while iron flux facilitates the execution of ferroptosis, zinc-dependent signaling potentially acts as a stabilizer of the endoplasmic reticulum and NF-κB pathways. Concurrently, a redox and metabolic buffering module, represented by *KEAP1* and *SAT1*, functions as a metabolic gatekeeping system where KEAP1 restrains NRF2-driven antioxidant responses and SAT1-mediated polyamine catabolism potentially serves as a metabolic sensor for cellular stress. Finally, an inflammatory gating module involving *TNIP1* and *TP53* is hypothesized to set the critical molecular threshold governing when an immune-mediated insult transitions from recoverable cellular stress to irreversible regulated cell death (22–32).

Immune infiltration profiling further supports this model. In ANCA-associated nephritis, enrichment of CD4^+^ T cells and monocytes reflects sustained inflammation, consistent with *SLC40A1*- and *TNIP1*-mediated regulation of iron and NF-κB signaling. In FSGS, elevated macrophages and CD8^+^ T cells imply cytotoxic and reparative immune interactions driven by iron metabolism and oxidative stress (33, 34). In MCD, increased B cells and monocytes, together with *KEAP1* and *SLC39A7* expression, indicate redox-dependent immune activation contributing to podocyte injury. In MN, associations of *TP53* and *TNIP1* with macrophages and T cells underscore immune-complex–driven oxidative injury. Collectively, these findings outline a convergent molecular landscape in which diverse immune-mediated stresses—whether driven by autoantibodies, complement activation, or podocyte-specific injury—amplify membrane vulnerability through the coupling of redox signaling and cell death pathways (35–41).

Conceptually, we interpret the “membrane vulnerability” framework as a molecular threshold system. In this proposed hypothetical model, the identified eight-gene axis does not merely correlate with injury but rather characterizes the intrinsic resilience of the glomerular filtration barrier. For instance, when KEAP1-mediated antioxidant capacity is compromised and iron export via SLC40A1 is dysregulated, the threshold for initiating ferroptosis or necroptosis is hypothesized to be significantly lowered. This provides a new interpretive depth for why clinically distinct immune insults—ranging from ANCA-driven inflammation to membranous nephropathy-related complement activation—may converge on a common pathological outcome: membrane destabilization and subsequent proteinuria. These processes disrupt podocyte and endothelial membranes, manifesting clinically as proteinuria and declining filtration. The identified gene panel thus not only provides preliminary molecular insight but also carries potential predictive and prognostic relevance. Interventions aimed at restoring NRF2 signaling, normalizing iron flux, or stabilizing polyamine metabolism could strengthen membrane resilience and complement current immunosuppressive therapies. It is important to emphasize that our proposed “membrane vulnerability” model represents a common terminal stage of injury rather than a shared etiology. For example, in ANCA-associated nephritis, the disruption of membrane regulation may be secondary to intense inflammatory cell infiltration, whereas in MN, it may result from subepithelial immune-complex-driven oxidative stress. By identifying the core eight-gene axis, we define a network that characterizes the cell’s baseline susceptibility to injury, helping to explain why glomerular damage persists across clinically distinct disease types.

Several limitations should be noted. First, the healthy control group in the primary dataset is relatively small compared with the immunonephropathy cohorts, which may introduce statistical bias. Although we applied robust machine-learning approaches and independent experimental validation to mitigate these concerns, the results should be interpreted with caution. Second, the current study relies on transcriptomic correlations and descriptive protein expression and lacks direct functional evidence from knockdown or overexpression assays to establish causal regulatory mechanisms. Third, our *in vivo* experiments were performed exclusively in male mice to reduce variability related to sex hormones and the estrous cycle; therefore, potential sex-specific differences in immune responses and renal injury could not be evaluated and should be addressed in future studies including female animals.

Future research should focus on two primary objectives. First, large-scale prospective clinical cohorts are required to further validate the diagnostic accuracy and prognostic stability of this eight-gene signature in diverse populations. Second, rigorous mechanistic studies are essential to elucidate the precise causal roles of these genes in modulating membrane integrity and regulated cell death pathways within podocyte and endothelial cell environments.

## 5 Conclusions

This study identifies dysregulated membrane homeostasis and regulated cell death pathways—specifically necroptosis, pyroptosis, and ferroptosis—as convergent mechanisms of injury across various immunonephropathy subtypes. Through an integrated machine-learning approach, we established a robust eight-gene diagnostic signature (*IPMK*, *TP53*, *SLC40A1*, *NCOA4*, *SLC39A7*, *KEAP1*, *TNIP1*, and *SAT1*) that effectively characterizes glomerular dysfunction and associated immune microenvironment alterations. These characteristic genes correlate significantly with clinical parameters such as eGFR and proteinuria, reinforcing their potential as reliable biomarkers for disease assessment and progression. Overall, our findings offer a novel molecular framework for understanding glomerular vulnerability and provide a foundation for developing targeted precision therapies in immune-mediated kidney diseases.

## Data Availability

The datasets presented in this study can be found in online repositories. The names of the repository/repositories and accession number(s) can be found in the article.
